# The Relationship Between Leadership Behaviors and Volunteer Commitment: The Role of Volunteer Satisfaction

**DOI:** 10.3389/fpsyg.2020.602466

**Published:** 2020-11-30

**Authors:** Paula Benevene, Ilaria Buonomo, Michael West

**Affiliations:** ^1^Department of Human Sciences, Libera Università Maria Santissima Assunta (LUMSA) University, Rome, Italy; ^2^Department of Organisation Work and Technology, Lancaster University Management School, Bailrigg, United Kingdom

**Keywords:** leadership, volunteer satisfaction, affective commitment, organizational learning, non-profit management, non-profit

## Abstract

Despite the relative scarcity of studies on the impact of leadership styles on satisfaction and commitment of volunteers within non-profit organizations, this relationship plays a crucial role in fostering sustained volunteerism and volunteers' well-being. A questionnaire was administered to more than 200 volunteers involved in delivering social services in non-profit organizations from Central and Northern Italy. The questionnaire contained the Volunteer Satisfaction Index, the sub-scale on Affective Commitment of the Organizational Commitment Scale, and two sub-scales of the Key Leadership Behaviors, namely: Helping people to grow and lead, and Enabling learning and innovation. Socio-demographic data were collected as well. Findings revealed that leaders' actions oriented toward the enablement of learning and innovation have an effect on volunteers' affective commitment, through the full mediation of volunteer satisfaction. Leaders' actions oriented toward the growth and empowerment of volunteers, instead, did not show significant relationships with volunteer satisfaction and affective commitment.

## Introduction

### The Relevance of the Non-Profit World

Non-profit organizations (NPOs) are organizations committed to promoting the well-being of individuals, communities, and society through the delivery of their services (Benevene and Cortini, 2010; Kong and Ramia, 2010; Dal Corso et al., 2019). NPOs are defined as private, independent, self-governed organizations, whose profits are not distributed to individuals or owners of stakeholders, but reinvested in the organizational mission, namely creating social value and contributing to general welfare (Salamon and Anheier, 1992; Bahmani et al., 2012).

All NPOs rely—partially or totally—on volunteers to deliver their services and carry out other tasks, such as office work, fundraising, and event organization to mention a few (Salamon and Anheier, 1992; Benevene and Cortini, 2010; Jiménez et al., 2010; Bahmani et al., 2012; Dal Corso et al., 2019). If all those who engaged in volunteering activities formed a country–the so-called “Volunteer Land” –this would be the ninth most populous country in the world, behind Russia and Bangladesh. In Europe alone, there are about 94 million people engaged in volunteering (Salamon et al., 2013). As far as the Italian context is concerned, the volunteer rate is 12.6% among the adult population (i.e., more than 6 million people)—out of which 7.9% operate in an organized context (ISTAT, 2019).

Thus, volunteers represent a crucial factor in the non-profit world. Their attraction, retention, and management are among the most strategic and challenging actions for NPOs (Anheier and Salamon, 2006; Salamon et al., 2013; Nencini et al., 2016; Alfes et al., 2017). In fact, volunteers' management requires taking into consideration the differences between them and employees: unlike paid staff, volunteers can choose the NPOs they prefer and are not bounded by a legal contract, assigning them weekly hours of duty, tasks, and responsibilities; they are not selected or rewarded on the basis of their professional competences and skills; they choose freely when to start participating in the NPO's activities and are free to leave whenever they will (Salamon and Anheier, 1992).

Most NPOs struggle to maintain their volunteers' engagement in the long term, although the number of people deciding to engage in volunteering activities is continually increasing and volunteers tend to continue volunteering over the course of their life, whether it is in the same organization, or in a different one (Garner and Garner, 2011). In this respect it has to be stressed that sustained volunteerism is an important component of the NPOs' organizational performance not only because these organizations need volunteers to carry out many of their activities, but also because long-term volunteering generates a better trained, more experienced, and more highly skilled volunteer base (Fairley et al., 2013).

Current research on sustained volunteerism suggests that when leaders are perceived positively, volunteers are more likely to be retained (Catano et al., 2001; Avolio et al., 2004; Richardson and Vandenberg, 2005; Rowold and Rohmann, 2009; Senses-Ozyurt and Villicana-Reyna, 2016). Despite this, little is known about the role of leadership styles on sustained volunteerism.

### Theoretical Basis of Sustained Volunteerism

Sustained volunteerism is commonly explained in light of the functional theory (Clary et al., 1998) and the social exchange theory (Blau, 1964).

The functional theory developed by Clary et al. (1998) and Clary and Snyder (1999) posits that volunteering provides opportunities to satisfy personal needs and drives. Clary and Snyder (1999, p. 157) identified six main motives pushing people to engage in voluntary work: values (the opportunity to express their values in the actions taken); understanding (the opportunity to learn or exercise skills that are often unused); career (the opportunity of professional growth through the acquisition of skills and knowledge useful for one's career path); social (the opportunity to strengthen one's own social relationships); enhancement (the opportunity to grow and develop psychologically through volunteer activities); protective (the opportunity to reduce negative feelings, such as guilt, or to address personal problems). Individuals may look for fulfillment of different motives in performing their volunteer activities and tasks. In other words, “different people engage in the same volunteer activity but do so to fulfill different motives” (Clary and Snyder, 1999, p. 156). When volunteers satisfy their motivations through their specific experience within the organization, they achieve higher performance, and greater satisfaction for the activities carried out, which in fact constitute relevant predictive factors for the decision to start and continue volunteering (Clary et al., 1998). The other theoretical basis of sustained volunteerism is offered by the Social Exchange Theory (Blau, 1964). According to this theory, volunteers decide to join an NPO assessing the cost-benefit balance due to their involvement in the NPO's tasks. It is likely that the higher the benefits perceived as a result of their work, the longer the commitment in the volunteering activity (Blau, 1964).

Apart from theoretical underpinnings, some authors pointed out that volunteering is a long-term planned behavior, within a dynamic process where various factors intervene (Omoto and Snyder, 1995; Penner, 2004). Thus, over time, the variables that come into play in leading someone to become a volunteer tend to change or take on a different weight from those that determined the initial choice. The shift from the initial motivations is somehow inevitable: after the first phase defined as “honeymoon,” where the volunteer is full of enthusiasm and desire to be engaged in the activities, a new phase takes place, connotated by a more realistic knowledge of the organization, based on the direct experience developed within the organization itself. The “post-honeymoon” phase necessarily bears feelings of disillusionment, since the idealization of the first months is replaced by the awareness of the critical aspects of the organization (Wymer and Starnes, 2001).

In this second phase, it may happen that the benefits and rewards obtained by volunteering may not be sufficient to compensate for their costs in terms of time, money, and personal resources required to perform the voluntary work. Thus, this new understanding carries the risk of leading the volunteer to leave the organization, if the critical factors are not counterbalanced by other positive factors generated by the actual experiences of volunteering (McCurley and Lynch, 1996).

In other words, the actual experience of volunteering changes the initial motivations of the volunteers (Snyder et al., 1999), either positively or negatively. These modifications indicate that the leadership of NPOs plays a pivotal role in providing support to the volunteers' choice to stay, shaping their experiences through effective managerial practices and choices (Umezurike, 2011; Senses-Ozyurt and Villicana-Reyna, 2016; Benevene et al., 2018). NPOs' leaders are, in fact, in charge of molding the operational activities of every volunteer; they hold responsibility for providing volunteers with positive organizational activities and experiences, which may compensate for the negative factors associated with volunteering.

### Leadership Style and Its Impact on Volunteers' Outcomes (Satisfaction and Commitment)

The knowledge about the impact of leadership styles on volunteers' behaviors is still far away from being fully explored. Whereas, on the one hand, it is well-known in the literature that NPOs' positive leadership is linked with sustained volunteering (Catano et al., 2001; Avolio et al., 2004; Richardson and Vandenberg, 2005; Rowold and Rohmann, 2009; Senses-Ozyurt and Villicana-Reyna, 2016; Yahaya and Ebrahim, 2016). on the other hand, many of the studies carried out on the outcomes of leadership styles on the members of NPO did not make a distinction between paid staff and volunteers (Allen et al., 2018; Einolf, 2018; Li, 2019; Peng et al., 2020).

This is an important gap to fill, since the motivations of volunteers are different from those of paid staff, and the management of volunteers must be tailored to their needs and motivations.

Studies carried out among NPOs have proven that the quality of the leadership is a critical factor for the volunteers' satisfaction and commitment, which, in turn, affects their turnover, intention to stay, performance, and well-being (Catano et al., 2001; Avolio et al., 2004; Richardson and Vandenberg, 2005; Senses-Ozyurt and Villicana-Reyna, 2016; Yahaya and Ebrahim, 2016).

Among the relative paucity of studies on the impact of leadership styles on satisfaction and commitment of volunteers, the most explored construct is transformational leadership. Transformational leaders inspire and motivate their followers to look beyond self-interest and to work together to pursue a collective purpose (Burns, 1998). An early study by Catano et al. (2001) found out that transformational leadership was associated with volunteers' commitment to the organization as well as their will to stay in the organization. Later, Dwyer et al. (2013) showed that transformational leadership influences volunteer satisfaction. These findings were partially confirmed by Schneider and George (2011), in a study carried out among volunteers, where transformational leadership did not appear to predict commitment. However, it showed significant positive correlations with satisfaction and intention to stay, through the respectively partial or full mediation of empowerment. On the other hand, the same study highlighted that servant leadership, connoted by ethical behavior and concern for subordinates, was associated with member satisfaction, commitment, and intention to stay in the same NPO, through the full mediation of empowerment (Schneider and George, 2011; Greenleaf, 2019).

Servant leadership emerged as positively correlated with volunteers' satisfaction and organizational commitment, also in a study by Erdurmazli (2019). Oostlander et al. (2014) considered the autonomy-supportive leadership, characterized by the understanding and the acknowledgment of volunteers' perspectives, giving them opportunities for choice, supporting their individuals' competences, and encouraging personal initiative (Deci et al., 2001; Gagné and Deci, 2005). Their study concluded that volunteer satisfaction and motivation is positively linked with this type of leadership. More recently, a study carried out by Benevene et al. (2018) observed the impact of ethical leadership on volunteers. Ethical leadership is defined as “the demonstration of normatively appropriate conduct through personal actions and interpersonal relationships and the promotion of such conduct through two-way communication” (Brown et al., 2005, p.120). This study showed that ethical leadership is connected with job satisfaction and organizational commitment among volunteers and that these factors have a positive impact on volunteers' intention to keep serving in the same NPOs in the long run.

Another recent study considered two dimensions of the Key Leadership Behaviors, which refer to inclusive and shared leadership, able to generate a culture of compassion toward the members of their organization, as well as toward the beneficiaries and end-users of their services (The King's Fund, 2017). This instrument is composed of the sub-scales or dimensions (Create a sense of collective identity; Create direction and alignment around strategies and objectives; Develop and empower people; Enable collective learning; Encourage trust and cooperation; Ensure necessary resources are available; Helping to interpret the meaning of events; Nurture commitment and optimism; Organize and coordinate work efforts; Promote social justice and morality). The Key Leadership Behaviors is focused on actual behaviors utilized by leaders, rather than on the perceptions of the members of organizations, thus contributing to the understanding of the leadership process.

Evidence from a study carried out among a group of Italian volunteers that considered two dimensions of this instrument (i.e., “Creating a sense of collective identity” and “Encouraging trust and cooperation”), showed that they are positively associated with work engagement, which, in turn, is positively related to volunteer satisfaction. The relationships between the two dimensions considered and volunteer satisfaction were found to be fully mediated by work engagement and to have an impact on volunteers' intentions to stay in the same organization (Dal Corso et al., 2019). These results seem promising.

In fact, the Key Leadership Behaviors were originally developed to be administered among healthcare systems, that is, among members of organizations offering people-oriented service. Therefore, it is possible to suppose that this instrument might be particularly suitable in offering interesting insights on the relationship between NPOs' leaders and volunteers involved in social services. It has to be stressed, in fact, that none of the leadership scale was developed to be administered among volunteers, but rather to paid staff.

Thus, in the present study two other dimensions of the Key Leadership Behaviors were taken into consideration, namely: “Helping people to grow and lead” and “Enabling learning and innovation,” “Helping people to grow and lead” refers to the construct of the empowering leadership, so that empowered followers can fully respond to a shared leadership. According to the Key Leadership Behavior, empowering leaders foster autonomy and build self-confidence and personal growth of their followers, in line with Menon's definition of empowerment (Menon, 1999, p.161), which is a “cognitive state characterized by a sense of perceived control, competence, and goal internalization.” Empowerment has emerged among both for-profit and non-profit organizations to play a relevant role in determining positive outcomes: empowered members of organizations report greater self-efficacy, which, in turn, fosters higher levels of satisfaction, commitment, effectiveness, and high performance (Kark et al., 2003). According to Spreitzer and Sonenshein (2004), empowerment involves values related to work goals, sense competence and autonomy, and perceived impact on organizational outcomes by means of one's own actions. Thus, empowerment correlates significantly with satisfaction with the activities performed, affective commitment, job satisfaction, and improved performance both among paid staff and volunteers (Galindo-Kuhn and Guzley, 2001; Choi et al., 2016). Nonetheless, the effects of a leadership able to empower volunteers are still poorly explored (Schneider and George, 2011). The relevance of this subscale of the Key Leadership Behaviors for volunteers can be grounded theoretically on the function of enhancement, according to the functional approach of Clary et al. (1998). Empowering leaders, in fact, may offer the opportunity of personal development (Anderson and Moore, 1978) and satisfaction related to personal growth and self-esteem (Jenner, 1982) through volunteering.

The other dimension considered, “Enabling learning and innovation,” refers to the involvement of each member of the organization in continuous learning, sharing, and generating new organizational knowledge, in order to reach better performance and higher quality services.

The relevance of the dimension “Enabling learning and innovation” refers to the function of understanding, according to the functional approach of Clary and colleagues (Clary et al., 1998; Clary and Snyder, 1999). Leaders who promote reflexivity and the sharing of individual knowledge also promote a deeper understanding of the problems that volunteers are dealing with through their actions, a better knowledge of the social environment where they operate and intervene, as well as sustaining their motivation to keep on volunteering.

Learning and skills development are common benefits of volunteering (Green and Chalip, 2009; Viel-Ruma et al., 2010), but, to the authors' knowledge, the issue of a leadership style promoting learning has been scarcely addressed in the context of volunteering. More precisely, Wisner et al. (2005) found that a very strong predictor of sustained volunteering is encouraging volunteers to reflect and learn on their work since this is a “way to help volunteers make sense of their experiences—both positive and negative—as they help to accomplish the organization's mission” (Wisner et al., 2005, p. 148). As Einolf (2018, p.159) points out, reflecting and learning “provides volunteers with an opportunity to think consciously about their experiences with others, to examine their own values and beliefs and to develop problem-solving skills.”

### Satisfaction and Organizational Commitment as Key Factors of Volunteers' Management

Studies on the outcome of effective leadership on volunteers' retainment, performance, and well-being took into consideration mainly two main constructs: satisfaction with the activities performed, and organizational commitment (Einolf, 2018).

This approach replicated the previous studies carried out in the managerial field, which have proven the strong link between organizational commitment and job satisfaction which, in turn, are highly associated with reduced absenteeism, low intention to quit, work effort and higher performance (Meyer et al., 2002; Park and Kim, 2009; Vecina et al., 2012).

As far as organizational commitment is concerned, Allen and Meyer (1990) developed a Three-Component Model composed of: affective commitment (referring to an emotional attachment to, identification with, and involvement in the organization); continuance commitment (referring to the perceived costs associated with leaving the organization); and normative commitment (referring to the perceived obligation to remain in the organization) (Meyer et al., 2002, 2006). Despite the fact that this concept was initially conceived to be used in the for-profit milieu, many of the studies carried out among NPOs observed organizational commitment, especially the facet of affective commitment when approaching both paid staff and volunteers or just volunteers. This dimension, in fact, is possibly considered the most effective in capturing the strength of the relationship between the volunteers and their organization (Stephens et al., 2004; Bang et al., 2013; Rodell et al., 2017; Ward and Greene, 2018). Thus, we aimed to verify the following hypotheses:

H1: Leaders' actions aimed at developing and empowering volunteers influence volunteer affective commitment;
H2: Leaders' actions aimed at enabling learning and innovation in volunteers influence volunteer affective commitment.

With regard to job satisfaction, this construct represents the extent to which people like or dislike their job (Spector, 1997). It has to do with how people feel about their job, the combination of positive or negative feelings that workers have toward their work. It is described as a set of beliefs and affects related to the daily work experience (Mowday et al., 1979). Similar studies on volunteers' affective commitment, as well as studies on employees and volunteers showed the strong relationship between satisfaction for the activities performed in one's own organization and intention to stay (Wisner et al., 2005; Vecina et al., 2009; Garner and Garner, 2011; Waters and Bortree, 2012; Nencini et al., 2016; Okun et al., 2016).

However, as Vecina et al. (2009) noted, the construct of job satisfaction as it is used among for-profit organizations or paid staff does not fit well with volunteers. Volunteer satisfaction is not merely the evaluation of how individuals feel about their organizational role, as for job satisfaction among paid workers (Spector, 1985). Unlike paid staff, volunteers do not find their satisfaction for the activities performed in career advancements, monetary recognition or benefits, or recognition of their professional skills. On the contrary, volunteers' satisfaction is a combination of several beliefs and affects the volunteer feels toward the NPO. Such beliefs include the extent to which the volunteering experience is consistent with personal values, whether their volunteering activities are perceived as useful, and whether they feel recognized and valued by the NPO.

Thus, Vecina et al. (2009, 2012) developed the Volunteer Satisfaction Index, identifying three peculiar facets in volunteer satisfaction: (1) satisfaction with their motivation to volunteer, (2) satisfaction with the tasks performed, and (3) satisfaction with the management of the NPO in which the volunteer operates. Their studies confirmed the association between volunteers' satisfaction and intention to stay as volunteers (Vecina et al., 2009, 2012). For the purpose of this study, the Volunteers Satisfaction Index was used., in order to verify the following hypotheses:

H3a: Leaders' actions aimed at developing and empowering volunteers are linked to volunteer satisfaction;
H3b: Leaders' actions aimed at enabling learning and innovation in volunteers are linked to volunteer satisfaction.

### The Mediating Role of Volunteer Satisfaction

Satisfaction and affective commitment of volunteers are both related to their well-being, performance, and intention to stay. However, while satisfaction is more determinant for newer volunteers, affective commitment is more crucial for veteran volunteers (Chacón et al., 2007; Vecina et al., 2012). This happens because volunteers' satisfaction is more linked to the first phase of the actual experience within the organization, and more subject to change, while affective commitment is built over time and tends to be more stable, being built over a more factual knowledge of the NPOs where volunteers operate (Mowday et al., 1979; Jiménez et al., 2010). In fact, the primary difference between these constructs relies on the stability of beliefs and affects related to them. Thus, somehow, satisfaction acts as a precursor of affective commitment in the volunteering experience, which, in turn, promotes sustained volunteerism (Chacón et al., 2007). Satisfied volunteers have higher chances to become more committed to the NPO over time (Jiménez et al., 2010; Cady et al., 2018). It seems, indeed, that satisfaction for the volunteering experience protects volunteers from the strain occurring from their activities and, at the same time, enhances the affective commitment toward the organization's mission and objectives, when a more realistic knowledge of their organization has been developed (Chacón et al., 2007).

Consequently, the authors also developed the following hypothesis:

H4: Volunteer satisfaction is linked to volunteer affective commitment.

Since volunteers' satisfaction is positively associated with their affective commitment, and both are linked with sustained volunteerism, effective NPO management needs to endorse leadership styles and strategies that generate volunteer satisfaction and affective commitment. This link is a crucial factor in guaranteeing the quality and the sustainability of their organization's activities.

Based on the functional theory of Clary et al. (1998) and Clary and Snyder (1999) it is possible to hypothesize that leaders who promote learning and understanding among those who freely devote their time and energies in the NPOs' activities, as well as leaders who are able to empower and support individual growth, are likely to offer a proper answer to the volunteers' needs and expectations, thus responding to the drives that push them toward volunteering (Chacón et al., 2007; Jiménez et al., 2010). The satisfaction with the volunteers' personal motivations to volunteer, together with the satisfaction with the tasks performed and with the management of the NPO, would lead to greater volunteer satisfaction and, in turn, would generate affective commitment.

Thus, the following hypotheses were developed:

H5a: Volunteer satisfaction mediates the relationship between leaders' actions aimed at developing and empowering volunteers and volunteer commitment
H5b: Volunteer satisfaction mediates the relationship between leaders' actions aimed at enabling learning and innovation and volunteer commitment.

### Key Contributions of This Study

This work aims to partially fulfill the gap regarding the role of leadership behaviors on volunteer retention. More specifically, building on the theoretical framework of the three-stage model of volunteers' duration of service (Chacón et al., 2007), this work aims to deepen the knowledge on the relationship between two leadership behaviors with the constructs tackling the first two stages of Chacon and colleagues' model, namely volunteer satisfaction and commitment. Furthermore, such a model would allow testing some aspects of the functional approach to volunteerism (Clary et al., 1992, 1998), namely enhancement and understanding.

**Figure 1:**
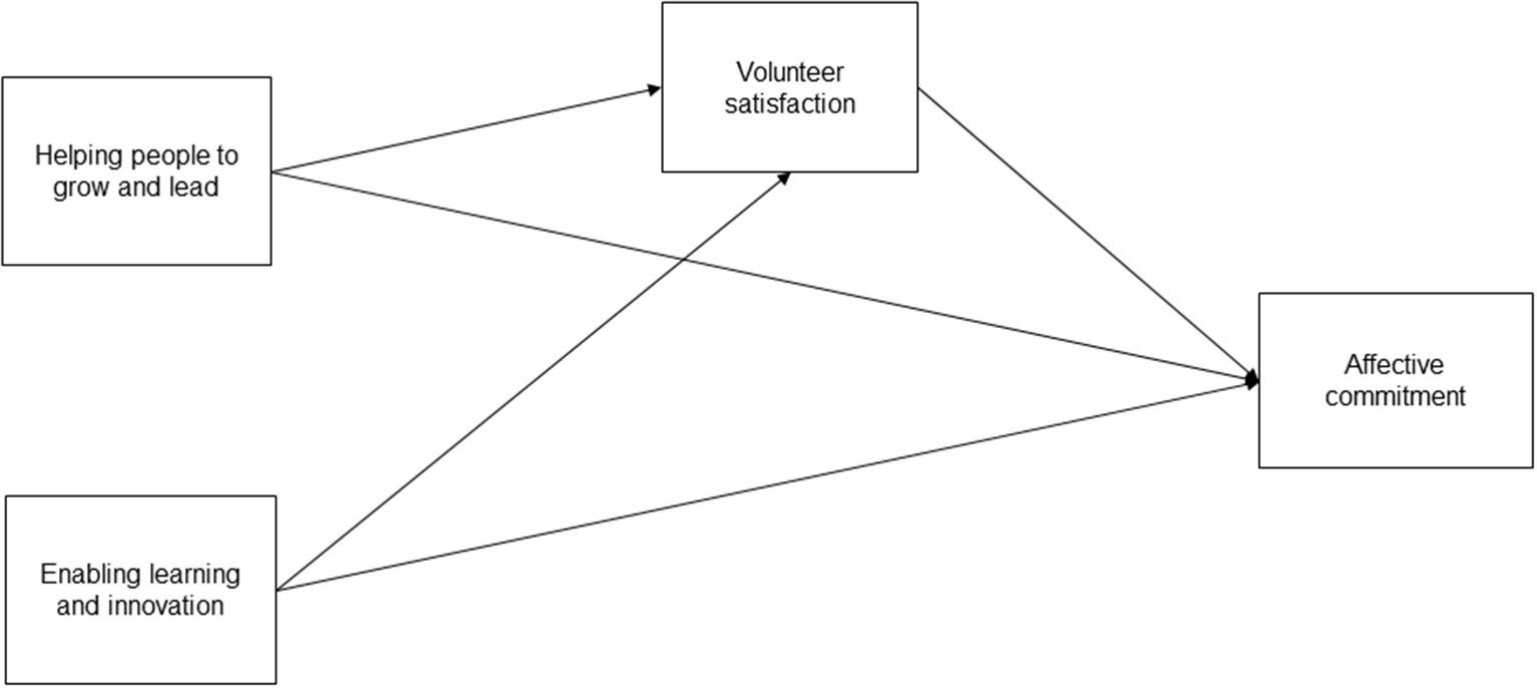
Theoretical model.

Overall, the proposed model, shown in Figure 1, verifies the mediating role of volunteer satisfaction in the relationship between leadership behaviors, namely actions oriented toward the enablement of learning and innovation and actions oriented toward the growth and empowerment of volunteers, and the volunteer commitment.

## Materials and Methods

### Participants and Procedure

Two hundred and twenty-four volunteers in health and social service-related NPOs (52.2% female) from Central and Northern Italy took part in this study. Their ages ranged from 14 to 76 (*M* = 38.42 years, SD = 16.24). Regarding educational level, 39.5% of participants have a high school degree, 31.2% an elementary or junior high school degree, 22.1% a graduate degree, and 7.2% a post-graduate degree. Regarding their occupational status, 26.2% were students, 22.1% employees, 12.8% freelancers, 12.4% retirees, 9.2% homemakers, 8.2% unemployed, 5.1% workmen, 1.5% teachers, 1.5% executives, and 0.5% merchants. Finally, regarding the duration of their service as volunteers, most of the participants (69.7%) had been a volunteer for one year or more, 19% for 6–12 months, 11.3% for <6 months. The participant volunteers constitute a convenience sample, not representative of the entire population of Italian volunteers.

Data were gathered by the research group at the end of NPOs board meetings. More specifically, by the end of the meeting a brief presentation of the research and its scope was given by one of the researchers, with the aim of informing the volunteers about the chance to take part, anonymously and voluntarily, in this study. All the volunteers willing to participate in the research were administered a copy of the protocol in an individual setting. This sampling strategy could have excluded volunteers not participating in the meeting during the established dates. At the same time, the authors preferred a one-day gathering to avoid the use of mixed gathering method (e.g., paper-pencil and online data gathering), or the influence of concomitant events between eventual multiple gathering sessions. The entire process was anonymous. Participants took part in the study after having received written information on Italian privacy regulations and having signed informed consent. The presentation of the study from an independent research group (and not from a NPO manager or employee), the provision of an individual setting to complete the protocol, and the anonymity and confidentiality of the procedures regarding data gathering and informed consent signing were the measures implemented to address a potential social desirability bias.

The research was conducted following the APA's ethical principles and code of conduct (APA, 2017). When an Italian validation was not available, the original versions of questionnaires were initially translated from English or Spanish into Italian and then back-translated into English or Spanish to check the alignment with the original versions.

### Measures

In order to assess the constructs under investigation, we used the following measures. Helping people to grow and lead and enabling learning and innovation variables were assessed with eleven items taken from Key Leadership Behaviors—The King's Fund Cultural Leadership Programme (The King's Fund, 2017). Each item was measured on a 5-point Likert scale ranging from 1 (strongly disagree) to 5 (strongly agree). The Cronbach's alpha is 0.89 for helping people to grow and lead and 0.88 for enabling learning and innovation. Sample items for the helping people to grow and lead scale (five total items) are: “(S)he supports the growth and development of team members”; “(S)he empowers team members to do the work in the way they think best”; “(S)he helps us to believe in ourselves to rise to new challenges.” Sample items for the Enabling learning and innovation scale (six total items) are: “(S) he motivates us to keep learning about ways of improving our services; “(S)he ensures we regularly take time to think through ways to improve our work”; “(S) he encourages us to reflect on what we can learn from times when work goes well.”

Volunteer satisfaction was assessed with the Volunteer Satisfaction Index (Vecina et al., 2009). The scale has 18 items, measured on a ten-point Likert scale ranged from 1 (I totally disagree) to 10 (I totally agree). The items are combined to provide three subscales: satisfaction with methods, satisfaction with tasks, and satisfaction with organizational management. The Cronbach's alpha for all the items is 0.90. Sample items are: “My volunteering allows me to express my personal values,” “The tasks that I perform are very useful.”

Affective commitment was assessed with six items from the Organizational Commitment Scale (Allen and Meyer, 1990). Each was measured on a 7-point Likert Scale, ranging from 1 (I totally disagree) to 7 (I totally agree). The Cronbach's alpha is 0.92. Sample items are: “I really feel like that's the organization's problems” (reverse-scored), “I enjoy discussing my organization with people outside it.”

### Data Analysis

First, a Confirmatory Factor Analysis (CFA) (Kline, 2011) was performed in order to examine the measurement model with MPlus version 8 (Muthén and Muthén, 2017). To enhance the reliability and parsimony of our model, item parcels were created for ‘Helping people to grow and lead' and “Enabling learning and innovation” (10 items) and “Affective Commitment” (six items). Each factor was defined by two parcels, to obtain fewer free parameters to estimate and to reduce the sources of sampling error (Little et al., 2002, 2013; Coffman and MacCallum, 2005), and each parcel was created by sequentially summing items assigned based on the highest to lowest item-total corrected correlations (Little et al., 2002, 2013; Coffman and MacCallum, 2005). The Robust Maximum Likelihood Approach (MLR) was used to deal with non-normality in data (Wang and Wang, 2012).

Next, the structural model (Model 1) was tested by using the structural equation modeling (SEM) approach (Kline, 2011). The model was conceptualized by using “Helping people to grow and lead” and “Enabling learning and innovation” (as measured by Key Leadership Behavior), “Volunteer satisfaction” (as measured by the Volunteer Satisfaction Index, as satisfaction with methods, tasks, and organizational management), and affective commitment (as measured by the Organizational Commitment Scale). We hypothesized both direct and indirect (through volunteer satisfaction) effects of “Helping people to grow and lead” and “Enabling learning and innovation” on affective commitment.

According to a multi-faceted approach to the assessment of the fit of the model (Tanaka, 1993), the following indices were used to evaluate the goodness-of-fit: the Chi-square likelihood ratio statistic, the Tucker and Lewis Index (TLI), the Comparative Fit Index (CFI), the Root Mean Square Error of Approximation (RMSEA), with its confidence intervals, and the Standardized Root Mean Square Residual (SRMR). We accepted TLI and CFI values >0.95 (Hu and Bentler, 1998), RMSEA values lower than 0.08 (Browne and Cudeck, 1992; Hooper et al., 2008) and SRMR values lower than 0.08 (Hu and Bentler, 1998; Hooper et al., 2008).

The following procedures of data exploration were applied: (a) uni- and multivariate outlier analysis (Mahalanobis's distance was set to *p* < 0.001) (Gath and Hayes, 2006); (b) score distribution analysis (skewness and kurtosis cut-off points were set to [−2; +2] (George and Mallery, 2003); (c) missing value analyses (missing values were skipped listwise) (Little, 1992). At the end of these procedures, we obtained the sample described above.

## Results

### Measurement Model

The measurement model showed a good fit to the data: $\chi_{\left(28\right)}^{2}$ = 29.882, *p* = 0.094, CFI = 0.983, TLI = 0.990, RMSEA = 0.043 (90% CI = 0.000–0.076, *p* = 0.588), SRMR = 0.037, confirming validity and distinguishability of the four theoretical constructs. The means, standard deviations, and correlations among the studied variables are presented in Table 1. As expected, affective commitment was associated with both Helping people to grow and lead (*r* = 0.444, *p* = 0.000) and Enabling learning and innovation (*r* = 0.475, *p* = 0.000), as well as to volunteer satisfaction (*r* = 0.496, *p* = 0.000). At the same time, volunteer satisfaction was correlated with Helping people to grow and lead (*r* = 0.474, *p* = 0.000) and Enabling learning and innovation (*r* = 0.481, *p* = 0.000). Socio-demographic and volunteering-related variables are not shown, as their associations with the variables of interest are not significant.

**Table 1:** Means, Standard deviations and Correlations among leader actions, volunteer satisfaction and volunteer commitment.

**Variables**	**Descriptive statistics**		**Correlations**			
	**M**	**SD**	**1**	**2**	**3**	**4**
1. Helping people to grow and lead	3.98	0.83	-			
2. Enabling learning and innovation	4.01	0.78	0.752^**^	-		
3. Volunteer satisfaction	7.86	1.59	0.474^**^	0.481^**^	-	
4. Affective commitment	4.11	0.80	0.444^**^	0.475^**^	0.496^**^	-

M, Mean; SD, Standard Deviation

** *p < 0.01*.

### Final Model

**Figure 2:**
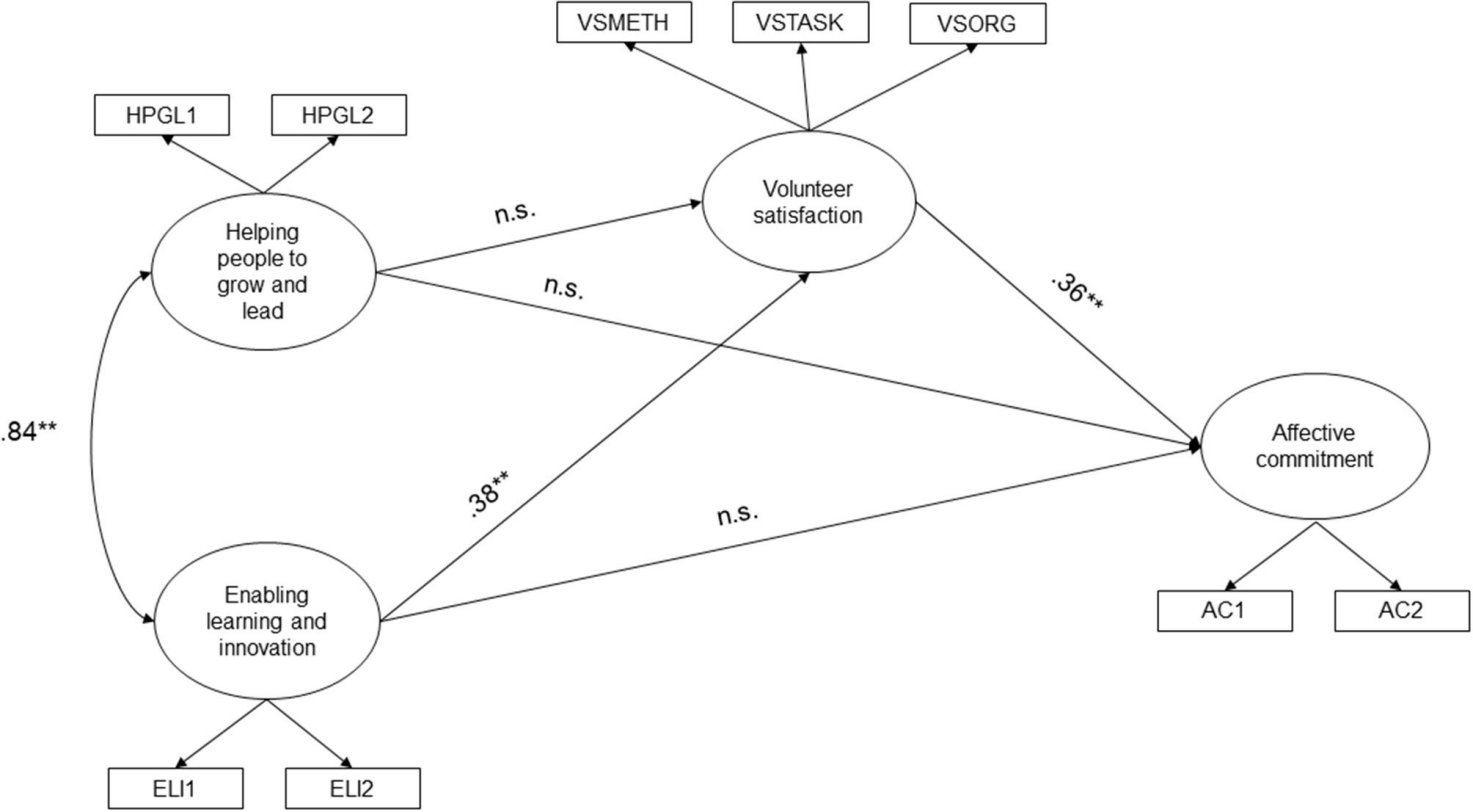
Final model. Standardized direct effects were reported. ** = *p* ≤ 0.01, n.s. = not significant.

Model 1 (Figure 2), hypothesizing both direct and indirect (through volunteer satisfaction) effects of Helping people to grow and lead and Enabling learning and innovation on affective commitment, proved to be an adequate fit to the data: $\chi_{\left(28\right)}^{2}$ = 29.882, *p* = 0.094, CFI = 0.983, TLI = 0.990, RMSEA = 0.043 (90% CI = 0.000–0.076, *p* = 0.588), SRMR = 0.037. Overall, Enabling learning and innovation was associated with Volunteer Satisfaction (*b* = 0.38, *p* = 0.010), but not with affective commitment (*p* = ns). Furthermore, Helping people to grow and lead did not show significant associations, neither with Volunteer satisfaction, nor with Affective Commitment. Finally, Volunteer satisfaction showed a significant direct effect on Affective commitment (*b* = 0.36, *p* = 0.000). The percentages of variance explained were 27.8% for volunteer satisfaction and 38.9% for affective commitment. Helping people to grow and lead and Enabling learning and innovation are significantly associated (*b* = 0.84, *p* = 0.001).

Volunteer satisfaction fully mediated the effect of Enabling learning and innovation on affective commitment (*b*_*DIRECT*_ = ns, *b*_*INDIRECT*_ = 0.14, *p* = 0.022; total indirect effect = 0.42, *p* = 0.016); Hayes (2013). At the same time, Helping people to grow and lead had no direct, nor indirect, significant effect on Affective commitment.

## Discussion

Managing volunteers is recognized as one of the most challenging tasks of NPOs' leadership, in the light of the high turnover rates of volunteers. At the same time, leadership style is a pivotal factor in volunteers' retention, productivity, and well-being (Garner and Garner, 2011). In fact, NPOs' leaders shape not only the organizational activities but also volunteers' behaviors through their actions, choices, and communication (Schneider and George, 2011). NPO leadership is responsible for keeping up volunteers' satisfaction and commitment, which are strong antecedents of volunteers' retention, performance, and well-being. Our findings suggested an involvement of leaders' actions oriented toward learning and innovation, but not of those oriented toward volunteers' growth and empowerment, in enhancing volunteers' satisfaction and commitment. Further sections will detail the theoretical and practical contribution of these results.

### Links With Previous Literature and Theoretical Contributions

Our study aimed at deepening the understanding of the association of leadership style with volunteer satisfaction and affective commitment, since these two constructs are linked to sustained volunteerism (Chacón et al., 2007). Thus, the objective of the present study was to observe how two subscales of the Key Leadership Behaviors (namely: “Helping people to grow and lead” and “Enabling learning and innovation”) are positively associated with volunteer satisfaction and affective commitment.

Research findings showed that leaders' actions oriented toward the enablement of learning and innovation have an effect on volunteer affective commitment, through the full mediation of volunteer satisfaction. Leaders' actions oriented toward the growth and empowerment of volunteers, instead, did not show significant relationships with volunteer satisfaction and volunteer affective commitment.

More precisely, our findings provided support for H3b (Leaders' actions aimed at enabling learning and innovation in volunteers are linked to volunteer satisfaction). These findings are in line with previous studies carried out almost exclusively in the for-profit organizations, showing a positive association between leaders' actions oriented at supporting collective learning and followers' satisfaction (Chang and Lee, 2007; Dirani, 2009; Bess et al., 2011; Razali et al., 2013; Dekoulou and Trivellas, 2015). These results might also be read in the light of the fact that volunteers do not often make full use of their professional skills to carry out their activities. Instead, they are urged to develop new skills through their direct volunteering experiences, such as team working, or communication or emotional regulation, just to mention a few of them. Thus, the development of new abilities and competencies might, at least in part, explain the positive association of volunteers' learning with their satisfaction. Again, this effect is theoretically explained by the function of understanding, according to the functional theory (Clary et al., 1998).

Similarly, H4 (Volunteer satisfaction is linked to volunteer affective commitment) and H5b (Volunteer satisfaction mediates the relationship between leaders' actions aimed at enabling learning and innovation and volunteer commitment) were also confirmed. The positive association of volunteers' satisfaction and their affective commitment, as well as the mediating role played by volunteer satisfaction in the relationship between positive behaviors and actions of the leadership on the one hand, and affective commitment on the other, are consistent with previous literature carried out among volunteers (Vecina et al., 2009; Benevene et al., 2018; Dal Corso et al., 2019). The results about the positive effects of leadership, enabling followers' collective learning and innovation on their affective commitment and satisfaction, are quite promising.

This finding deserves further attention, since creating workplace learning has proven to influence not only job satisfaction (Rowden and Conine, 2005; Iliopoulos et al., 2018; Ryu and Moon, 2019), but also job performance (Judge et al., 2001) and knowledge generation (de Grip, 2015). Promoting learning among the members became a strategic issue in the management of organizations since 1990 (Senge, 1991). According to Senge, an effective leader is required to be able to foster collective learning by catalyzing the whole organization around learning, rather than on the individual members (Senge, 1991, 2006). Being able to learn constantly has become a crucial factor of all organizations, private and governmental, for-profit, and non-profit, since this factor allows an organization to survive and grow, as well as to be able to cope with the challenges of a continuously changing environment. Learning is necessary to improve the services provided, to cope with new needs and new challenges, through a bottom-up approach. However, organization learning always starts from individual learning through critically re-thinking the activities performed, and then socializing the individual knowledge developed. In this way the knowledge of each member of an organization may be turned into organizational knowledge, according to the theory of the spiral of knowledge (Nonaka, 1991). The new knowledge may then be turned into innovation and improved service. Thus, the more the leader fosters organizational learning, the higher the organizational adaptability to community requests (Heifetz and Laurie, 1997; Kouzes and Posner, 2007). NPOs' leadership holds responsibility over promoting and sustaining this process, which has a two-fold outcome: volunteers' satisfaction which, in turn, is associated with affective commitments and higher organizational performance. In spite of the fact that this leadership dimension has been poorly observed before among volunteers and empirical data is by far scarce, this could be an interesting avenue for further study that would anchor leaders who operates in the direction of collective learning and volunteers' outcomes.

H2 (Leaders' actions aimed at enabling learning and innovation in volunteers influence volunteer affective commitment) was not confirmed, since our findings show no direct association of the dimension of leadership “Enabling learning and innovation” with the affective commitment of volunteers. Results from previous studies, carried out until now only among non-profit organizations and dealing with the relationship between learning and organizational commitment, are not always consistent. Several studies carried out also in non-Western countries proved the impact of learning on organizational commitment (Rose et al., 2009; Budihardjo, 2013; Lau et al., 2017). For instance, Jerez-Gómez et al. (2005) found out that learning behaviors increase organizational citizenship behaviors, job performance, job satisfaction, organizational commitment, belief in information, goal commitment, satisfaction with the leader, and low intentions to quit. Similarly, Kamali et al. (2017) pointed out a positive and direct relationship between organizational learning and organizational commitment of staff. Conversely, Suifan and Allouzi (2018) found no direct effect of staff learning on affective commitment.

Nonetheless, the association of leadership enabling collective learning ad innovation with affective commitment through the full mediation of job satisfaction might be read in the light of the “Three-stage model of volunteers' duration,” developed by Chacón et al. (2007). According to this model, sustained volunteering goes through different phases: the first one is the satisfaction with the initial motivations, the second one is the commitment with the organization they serve, and the third one is the role identification as volunteers. Thus, our findings seem to suggest the key role played by volunteers' satisfaction in generating their affective commitment, at least in the relationship between leadership enabling collective learning and innovation on one side, and affective commitment on the other.

Like H2, also H1 (Leaders' actions aimed at developing and empowering volunteers influence volunteer affective commitment), H3 (Leaders' actions aimed at developing and empowering volunteers are linked to volunteer satisfaction) and H5a (Volunteer satisfaction mediates the relationship between leaders' actions aimed at developing and empowering volunteers and volunteer commitment) were not confirmed by our study. To the authors' knowledge, this study is the first dealing with the effect of empowering leadership on volunteers' affective commitment and satisfaction. A number of studies carried out among for-profit and public organizations showed the positive association between this leadership style on the two considered variables, as well as on work engagement and psychological empowerment (Vecchio et al., 2010; Hassan et al., 2013; Amundsen and Martinsen, 2015; Kim et al., 2018; Kim and Beehr, 2020).

Interestingly, some studies showed the effects of empowering leadership on job satisfaction through either the full or partial mediation of other factors, such as the psychological empowerment (which refers to the perception of being empowered, through the dimensions of meaningfulness of the activities performed, competence, self-determination, and impact of one's own work) or the leader-member exchange relations (Albrecht and Andreetta, 2011; Hassan et al., 2013; Amundsen and Martinsen, 2015).

It might be hypothesized, then, that empowerment operates on job satisfaction through the mediation of other factors. Therefore, in the future, it would be interesting to explore the mediating role of psychological empowerment between the empowering leadership and volunteers' satisfaction or affective commitment. In other words, empowering leaders might not have an effect on the satisfaction and commitment of their followers if these do not perceive their own empowerment.

Another explanation for the lack of positive association of empowering leadership with volunteers' satisfaction and affective commitment might arise from a couple of previous studies carried out among volunteers, which proved that empowering leadership is positively associated with volunteers' engagement (Tuckey et al., 2013; Kang, 2016). From these studies, it emerged that empowering leadership develops volunteers' engagement through the improvement of their working conditions. In fact, according to Tuckey et al. (2013, p. 23) “empowering leadership optimized the combination of cognitive job demands and cognitive job resources for followers to achieve at work (a form of extrinsic motivation) and feel fulfilled (a form of intrinsic motivation). Thus, leaders who empowered their followers … created better working conditions for workers. The end result was an increase in engagement.”

These findings suggest that empowerment leadership has an effect on volunteers, generating positive feelings about their activities-related issues, and counteracting the effect of negative emotions, such as emotional exhaustion and cynicism. Empowering leadership may then promote positive work emotions among their followers, helping them to foster their personal resources. This means that empowering leadership has definitely a relationship with the positive feelings of volunteers about how they perceive their activities, while it might not be directly associated with the volunteers' satisfaction (that is: with their motivation to volunteering, or the tasks performed, or with the NPO in which the volunteer operates) or with their affective commitment with their organization.

The relationship between empowering leadership and affective commitment of volunteers is worthy of future research, since other leadership styles showed different results. Ethical leadership, for instance, has proven to be positively associated with volunteer affective commitment both directly and partially through the full mediation of volunteer satisfaction. From the study carried out by Schneider and George (2011), transformational leadership did not significantly predict affective commitment, unlike the findings of Erdurmazli (2019) on the relationship between servant leadership and affective and normative commitment.

Moreover, a further point has to be stressed: the study developed by Dal Corso et al. (2019) found out that two other dimensions of the Key Leadership Behaviors (namely: “Creating a sense of collective identity” and “Encouraging trust and cooperation”) are positively associated with job satisfaction, but through the full mediation of work engagement. On the other hand, our study on two other dimensions of the same instrument showed different results.

This is an interesting point to address, also in the light of the previous different results reached by studies that observed other types of leadership in their outcomes on volunteers. It might be hypothesized that some types or some dimensions of a specific leadership style could better suit volunteers than others (Spears, 1998; Erdurmazli, 2019). It could also be hypothesized that culture and contingencies might exert a role in determining the relationship between leadership style and volunteers' satisfaction and affective commitment or, more generally, with volunteers' outcomes. For these reasons, it would be worth to develop multilevel and cross-cultural studies. The volunteers reached by this study were all working in delivering social services. Perhaps other volunteers, committed with other tasks and volunteer environment (such as in a library or with firefighters), might show different outcomes (Waters and Bortree, 2012; Henderson and Sowa, 2018; Oh, 2019).

### Practical Implications

This work has interesting implications for NPO management. It shows the relevance of making NPOs' managers aware of their leadership style in retaining volunteers and promoting their well-being. First of all, this study shows the value of learning practices for volunteers. Having the opportunity to learn new things is one of the motivations for volunteering (Clary et al., 1998; Clary and Snyder, 1999), as well as one of the main benefits of being a volunteer (Green and Chalip, 2009; Viel-Ruma et al., 2010). Our findings show the importance of giving value to organizational learning processes, in terms of impact on volunteer satisfaction and commitment and, likely, on their retention. In order to achieve such outcomes, NPO managers should give more value to formal and informal knowledge creation and management processes. At the same time, the manager should be actively involved in the enablement of such practices, for example, by encouraging the team to assess and review practices, structures, and working styles, or implementing regular meetings aimed at improving the work. Overall, higher volunteer satisfaction, commitment, and retention would allow the NPO to perform better.

Secondly, it is interesting to note that volunteer satisfaction fully mediates the effect of promoting organizational learning. This implies that NPO managers should address volunteers' motivations and subsequent satisfaction levels, in order to verify whether and how the organization could contribute to improving them. Previous studies showed that when managers address efficaciously volunteer motivations, their volunteers are more likely to be highly satisfied with their work in the NPO (Schneider and George, 2011; Dwyer et al., 2013; Oostlander et al., 2014).

Thirdly, a number of studies addressed how personality traits may influence the decision to volunteer but, from a strictly managerial point of view, it is more productive to dwell on the organizational and managerial aspects than on dispositional or personality traits of volunteers since the former are the more directly controllable by the organization. In fact, the analysis of personality and dispositional traits could undoubtedly constitute a factor to be evaluated in the selection phase of new volunteers. However, very few organizations can afford to discard any volunteers based on their mismatch between these traits and organizational aspects (Elshaug and Metzer, 2001; Pushkar et al., 2002; Li et al., 2007; Van Vianen et al., 2008).

### Limitations

Firstly, this study is based on correlational data. Longitudinal, as well as qualitative, studies would allow researchers to better understand to what extent leaders supporting individual and collective needs promote volunteer satisfaction and engagement. Furthermore, considering the concerns regarding the existence of common method bias, and despite applying some suggestions from Conway and Lance (2010) (e.g., preservation of anonymity, removal of unengaged/outlier responses, testing each scale reliability and the general measurement model), we were unable to provide a multi-informant source for our data.

Secondly, some studies (Haivas et al., 2012; Oostlander et al., 2014) shed light on the chance that the collective dimensions of volunteering could not be as salient as shown in this paper or in the cited researches (Boezeman and Ellemers, 2009). More research is needed to clarify better the role of group and collectivity in the volunteering experience. More specifically, it could be useful to study specific kinds of NPOs and verify which leaders' actions are more valued by volunteers, according to NPOs' tasks and objectives. For example, it is plausible that NPOs oriented toward psychological assistance (e.g., helplines) could require more competence and a higher sense of empowerment and skills growth in volunteers when compared to less helping-oriented organizations.

## Data Availability Statement

The datasets for this article are not publicly available because of local legal and privacy restrictions (Italian Data Protection Code—Legislative Decree No. 196/2003). Requests to access the datasets should be directed to Paula Benevene, benevene@lumsa.it.

## Ethics Statement

Ethical review and approval was not required for the study on human participants in accordance with the local legislation and institutional requirements. The patients/participants provided their written informed consent to participate in this study.

## Author Contributions

PB, IB, and MW developed the research project and collected the data. IB conducted the analyses. PB and IB wrote the first draft of the paper. MW reviewed the paper. All authors contributed to the article and approved the submitted version.

## Conflict of Interest

The authors declare that the research was conducted in the absence of any commercial or financial relationships that could be construed as a potential conflict of interest.
