# Corrigendum: The Relationship Between Leadership Behaviors and Volunteer Commitment: The Role of Volunteer Satisfaction

**DOI:** 10.3389/fpsyg.2020.636558

**Published:** 2021-01-08

**Authors:** Paula Benevene, Ilaria Buonomo, Michael West

**Affiliations:** ^1^Department of Human Sciences, Libera Università Maria Santissima Assunta (LUMSA) University, Rome, Italy; ^2^Department of Organisation Work and Technology, Lancaster University Management School, Bailrigg, United Kingdom

**Keywords:** leadership, volunteer satisfaction, affective commitment, organizational learning, non-profit management, non-profit

In the original article, we neglected to include the funder LUMSA University, to Paula Benevene.

The authors apologize for this error and state that this does not change the scientific conclusions of the article in any way. The original article has been updated.

